# Development of Antibacterial Resin Composites Incorporating Poly(METAC) Clusters

**DOI:** 10.3390/ma17040896

**Published:** 2024-02-15

**Authors:** Tomoki Kohno, Haruaki Kitagawa, Ririko Tsuboi, Fan Deng, Hirohiko Sakai, Tingyi Wu, Yo-Shiuan Fan, Linghao Xiao, Satoshi Imazato

**Affiliations:** 1 Joint Research Laboratory of Advanced Functional Materials Science, Osaka University Graduate School of Dentistry, 1-8 Yamadaoka, Suita, Osaka 565-0871, Japan; 2 Department of Dental Biomaterials, Osaka University Graduate School of Dentistry, 1-8 Yamadaoka, Suita, Osaka 565-0871, Japan; 3 Department of Cariology, Restorative Sciences and Endodontics, University of Michigan School of Dentistry, 1011 N University Ave, Ann Arbor, MI 48109, USA

**Keywords:** antibacterial resin composite, METAC, quaternary ammonium compounds

## Abstract

This study examined the antibacterial effects and physical properties of a novel resin composite incorporating poly[{2-(methacryloyloxy)ethyl}trimethylammonium chloride] (poly(METAC)), a methacrylate cationic polymer comprising quaternary ammonium compounds (QACs). Resin composites incorporating poly(METAC) were fabricated by adding 6 wt.% METAC aqueous solution to a commercially available resin composite. The FE-SEM/EDS and Raman spec-troscopy analyses showed that METAC was assembled and polymerized in the resin composites after curing. The antibacterial effect was evaluated by inoculating Streptococcus mutans or Strepto-coccus sobrinus suspensions on the surface of cured resin composites, and the experimental resin composites incorporating poly(METAC) clusters exhibited bactericidal effects even after 28 days of ageing. The physical properties of the experimental resin composites were within the ISO-stipulated ranges. Newly fabricated resin composites containing the QAC-based poly(METAC) cluster ex-hibited long-term bactericidal effects against oral bacteria on their surfaces and demonstrated ac-ceptable physical properties for clinical use.

## 1. Introduction

The development of new-generation dental materials with antibacterial activity is a topic of considerable interest and ongoing research in the field of dentistry [1]. Among the several approaches available for imparting antibacterial effects to dental resins, one useful technology immobilizes antibacterial components on materials [1,2,3]. These materials exhibit a phenomenon known as “contact inhibition”, where bacteria that come into contact with the material surfaces are suppressed, without the release of any active components [1]. This technology contains the utilization of a polymerizable bactericide, such as resin monomers based on quaternary ammonium compounds (QACs) [2].

Imazato et al. reported the first polymerizable bactericide for dental materials, the antibacterial monomer 12-methacryloyloxydodecylpyridinium bromide (MDPB) [3]. MDPB was synthesized by combining alkylpyridinium (a QAC) with a methacryloyl functional group. The alkylpyridinium in the MDPB is responsible for its antibacterial effects, and the methacryloyl functional group can copolymerize with other monomers used for dental resins [1,3]. Bactericides immobilized via the polymerization of MDPB covalently bind to dental resins and cannot be released. Therefore, resin composites containing MDPB exhibit long-term inhibition against microorganisms on their surfaces [4]. However, the immobilized bactericide exhibits limited molecular movement and interferes with the structure of the bacterial surface through direct contact; therefore, its effect is essentially bacteriostatic rather than bactericidal [1]. To obtain bactericidal effects, the density of the immobilized QACs exposed on the outer surface must be high, as shown by quaternary ammonium polyethyleneimine (QPEI) nanoparticles [5]. Recently, we developed a novel strategy to increase the density of immobilized MDPB and enhance its antibacterial and antibiofilm properties by combining a surface-grafting technique with electron-beam (EB) irradiation [6]. The immobilization of antibacterial MDPB on dental resins via EB irradiation induced bacterial-membrane depolarization, thereby increasing membrane permeability and eventually causing celldeath. However, a technique for immobilizing high-density antibacterial components by incorporating antibacterial monomers into resin composites has yet to be developed.

**Figure 1:**
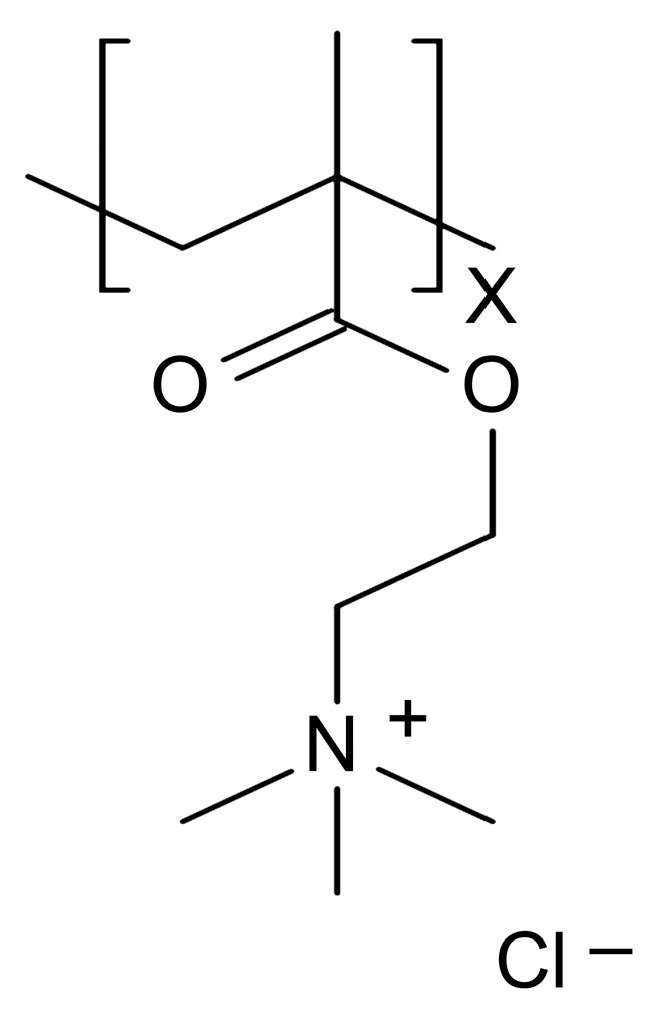
Structural formula of poly(METAC).

**Figure 2:**
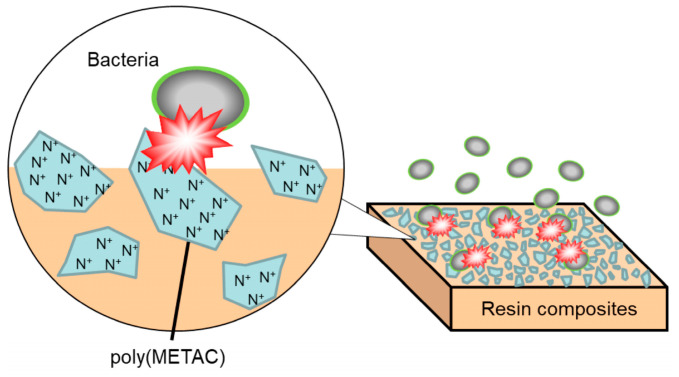
Schematic of antimicrobial activity of poly(METAC).

Poly[{2-(methacryloyloxy)ethyl}trimethylammonium chloride] (poly(METAC)) is a methacrylate-type cationic polymer that comprises QAC (Figure 1). METAC was reported to demonstrate antibacterial effects when polymerized alone via radical polymerization or when copolymerized with other types of monomers [7]. Considering the short alkyl chain length of METAC, the antimicrobial activity of poly(METAC) is probably derived from the charged nitrogen atom of quaternary ammonium, which concentrates the positive charge. In the dental field, adhesive resins or resin sealants containing the METAC monomer have been reported to demonstrate the contact inhibition of *Streptococcus mutans* on their surfaces [8,9]. However, the antibacterial effects of resin composites containing poly(METAC) have not been investigated. According to the emulsion principle, water-soluble METAC monomers agglomerate when incorporated into resin composites. Therefore, clusters of poly(METAC) may be formed in resin composites by aggregating and polymerizing METAC monomers. Resin composites incorporating poly(METAC) clusters were hypothesized to exhibit bactericidal effects on their surfaces because of the high density of charged nitrogen atoms in METAC (Figure 2). This study aimed to fabricate resin composites incorporating poly(METAC) clusters and evaluate their antibacterial effects.

## 2. Methods

### 2.1. Fabrication and Characterization of Resin Composites Incorporating Poly(METAC)

METAC was purchased from Tokyo Chemical Industry Co., Ltd. (Tokyo, Japan). An aqueous solution of METAC was prepared and mixed with commercial resin composites (GraceFil ZeroFlo, GC Corporation, Tokyo, Japan; hereafter denoted as GZ). The METAC concentration in the experimental resin composite (hereafter denoted as EX) was 6 wt.%. The compositions of the resin composites used in this study are summarized in Table 1. A mold (9 mm diameter, 2 mm thickness) was filled with the resin composite paste of EX. The surface was covered with celluloid strips and a glass slide, and both sides were cured using a light-activation unit (Alpha Light V; Morita, Kyoto, Japan) for 1 min each. The light intensity on the turntable of this unit was 35 mW/cm^2^. The resin disc was stored for 24 h at 25 °C and then polished using silicon carbide grinding paper (Buehler, Lake Bluff, IL, USA) from #120 to #1200. The samples were sterilized with ethylene oxide at 40 °C for 24 h. The EX and GZ surfaces were observed using a scanning electron microscope (SEM; TM3000, Hitachi High-Tech, Tokyo, Japan) at 5 kV. The elemental compositions of EX and GZ were analyzed using field-emission scanning electron microscopy/energy-dispersive spectroscopy (FE-SEM/EDS; JSM-F100, JEOL, Tokyo, Japan) at 10 kV.

Raman spectroscopy (inVia Raman Microscope, Renishaw plc, Wotton-under-Edge, UK) was used to measure the degree of conversion (DC) of poly(METAC) in the experimental resin composites. The METAC aqueous solution was measured and used as a reference Raman spectrum. The areas of the obtained spectra of carbon–carbon double bonds (1640 cm^−1^) and carbon–oxygen double bonds (1710 cm^−1^) were measured, and the rates of the carbon–carbon double bonds in poly(METAC) (PMC) and the METAC aqueous solution (MC) (R_PMC_ or R_MC_) were calculated as follows: R_PMC_ and R_MC_ (%) = 100 × (Area of C=C at 1640 cm^−1^ / Area of C=O at 1710 cm^−1^). Then, DC (%) was calculated as follows: DC (%) = (R_MC_ − R_PMC_) / R_MC_. Five specimens (n = 5) were prepared and used for the experiments.

To confirm that the unpolymerized METAC monomer was not released from the cured EX, the sample was stored in 10 mL of distilled water for 28 days at 37 °C while being shaken at 100 rpm. The METAC concentration in the eluate was measured using high-performance liquid chromatography (HPLC; Prominence; Shimadzu, Kyoto, Japan) using a reverse-phase column (Puresil 5µ C18; Waters, Milford, MA, USA). A mixture of acetonitrile and 5 mM phosphate-buffered saline (PBS; Wako, Osaka, Japan) containing 100 mM sodium perchlorate (70:30, *v*/*v*) was used as the mobile phase at a flow rate of 1 mL/min. Absorbance was recorded at 260 nm. 

### 2.2. Antibacterial Activity of Resin Composites Incorporating Poly(METAC)

An on-disc culture assay was conducted to evaluate the antibacterial effects of the non-leachable components immobilized in EX [10]. *Streptococcus mutans* NCTC10449 and *Streptococcus sobrinus* ATCC 33478 suspensions were adjusted to 1 × 10^5^ colony-forming units (CFU)/mL using brain–heart infusion broth (BHI; Becton Dickinson, Sparks, MD, USA). The *S. mutans* suspensions (50 μL) were inoculated onto cured EX discs; GZ was used as a control. After anaerobic incubation at 37 °C for 24 h under high-humidity conditions to prevent evaporation of the bacterial suspension, the inoculated discs were transferred to 9.95 mL of PBS (Wako, Osaka, Japan) and sonicated for 10 min in an ultrasonic bath operating at 37 kHz and 300 W to detach the bacteria. After ultrasonication, a SEM was used to observe that no bacteria were left on the specimens. The suspensions were serially diluted, and their aliquots were spread on BHI agar plates (Becton Dickinson). After anaerobic incubation at 37 °C for 24 h, the number of colonies was counted. Six specimens (n = 6) of each material were prepared and used for the experiments.

To evaluate the long-lasting antibacterial effects, the newly prepared cured discs were immersed in distilled water (500 µL) at 37 °C for 28 days and subsequently subjected to an on-disc culture assay. GZ was used as the control. Six specimens (n = 6) of each material were prepared and used for the experiments. To confirm the inhibitory effect of the eluate from EX on bacterial growth, the eluate (50 µL) after the immersion of EX for 28 days was collected and added to the *S. mutans* NCTC10449 or *S. sobrinus* ATCC 33478 suspensions (50 µL), adjusted to 2 × 10^5^ CFU/mL in BHI broth. After 24 h of anaerobic incubation, the number of colonies was determined as described above. GZ was used for comparison, and a bacterial suspension without any eluates (*w*/*o*) served as the control. Three specimens (n = 3) of each material were prepared and used for the experiments.

The cationic membrane-permeable fluorescent dye 3,3-dipropylthiacarbocyanin iodide (DiSC_3_(5)) was used to assess bacterial–membrane depolarization. An *S. mutans* suspension was prepared via incubation with 0.4 µM DiSC_3_(5) solution at 37 °C for 30 min and further incubation with 100 mM KCl for 1 h. Subsequently, EX or GZ discs (9 mm diameter, 2 mm thickness) were immersed in 500 µL of the prepared suspension. After incubation while shaking at 100 rpm for 3 h, 200 µL of the incubated suspension was placed in a well of a black microplate (Corning Inc., Corning, NY, USA). The fluorescence was measured at excitation and emission wavelengths of 622 and 670 nm, respectively, using a fluorescence detector (SH-9000 Lab Series; Corona Electric, Ibaraki, Japan). The fluorescence intensity after 24 h of incubation in the presence of EX or GZ was determined by subtracting the fluorescence value obtained by measuring the *S. mutans* suspension after 24 h of incubation in the absence of any materials from the initial fluorescence value. The experiment was repeated six times (n = 6).

### 2.3. Physical Properties of Resin Composites Incorporating Poly(METAC)

The flexural strength, curing depth, water absorption, and dissolution of EX were determined according to International Organization for Standardization (ISO) 4049: 2019 [11]. The flexural strength was evaluated using a three-point bending test with a tabletop testing machine (EZ-S, Shimadzu, Kyoto, Japan). The cured specimen was prepared using a metal mold (25 × 2 × 2 mm) and stored in water at 37 °C for 7 days. The loading force was applied to the specimens at a crosshead speed of 1 mm/min. Three specimens (n = 3) were prepared and used for the experiments. 

To measure the curing depth of EX, the resin pastes were filled in a stainless split mold with a cylindrical cavity (φ 4 mm × 6 mm) and vertically irradiated for 20 s using an LED-light curing unit (G-Light Prima-II Plus, GC Corporation, Tokyo, Japan) in contact with one end. The uncured paste was then removed using a spatula. The height of the cured resin was measured using a digital caliper (Absolute Digimatic Caliper, Mitutoyo Corporation, Kanagawa, Japan), and the height was divided by two to obtain the curing depth. Three specimens (n = 3) were prepared and used for the experiments.

To measure water absorption and dissolution, the cured EX (φ 15 × 1.0 mm^2^) was polished with fine sandpaper and then stored in a desiccator at 37 °C. The sample weights were measured after conditioning in a desiccator at 37 °C for 22 h and 23 °C for 2 h using an electric balance; the resulting accuracy was 0.01 mg. Subsequently, the specimens were immersed in a water bath at 37 °C for 7 days. The samples were then removed from the bath, dried with filter paper, and weighed. The water uptake was calculated, and the specimen was dried again until a constant weight was obtained, after which the dissolution amount was calculated. Three specimens (n = 3) were prepared and used for the experiments.

### 2.4. Statistical Analysis

Statistical analyses were performed using Statistical Package for the Social Sciences Statistics 25 (IBM Corp., Armonk, NY, USA). The homogeneity of variance was initially confirmed. The results of the quantification of bacterial cells were statistically analyzed using one-way analysis of variance (ANOVA) and Tukey’s honest significant difference (HSD) tests. The data pertaining to membrane depolarization were statistically analyzed using Student’s *t*-test. *p*-values below 0.05 were considered to indicate statistical significance.

## 3. Results

### 3.1. Fabrication and Characterization of Resin Composites Incorporating Poly(METAC)

**Figure 3:**
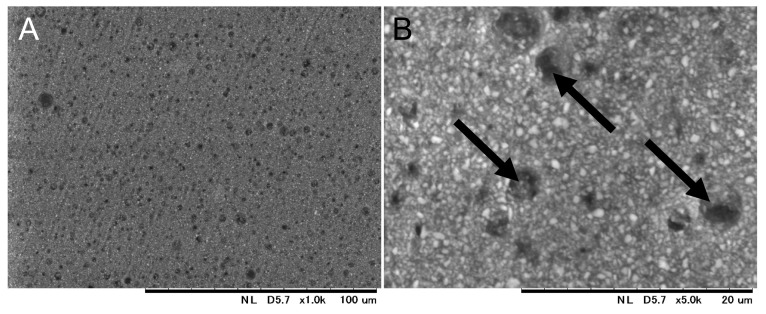
SEM image of the surface of cured EX: (**A**) low magnification; (**B**) high magnification. Arrow: poly(METAC).

**Figure 4:**
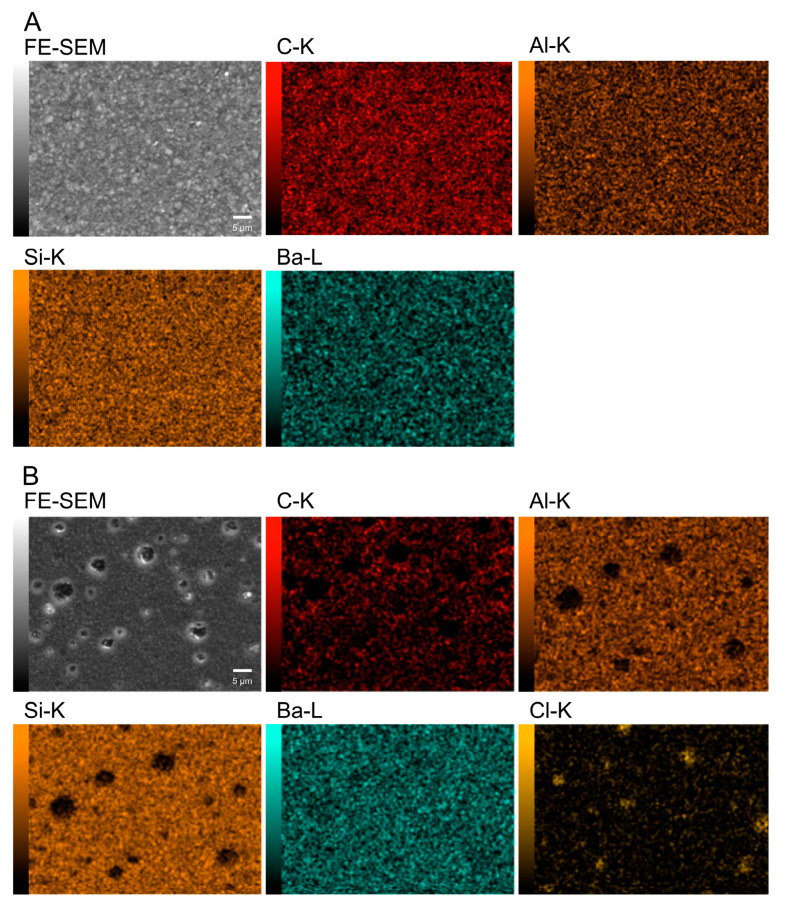
Elemental mapping image on (**A**) GZ and (**B**) EX surfaces obtained via EDS analysis.

**Figure 5:**
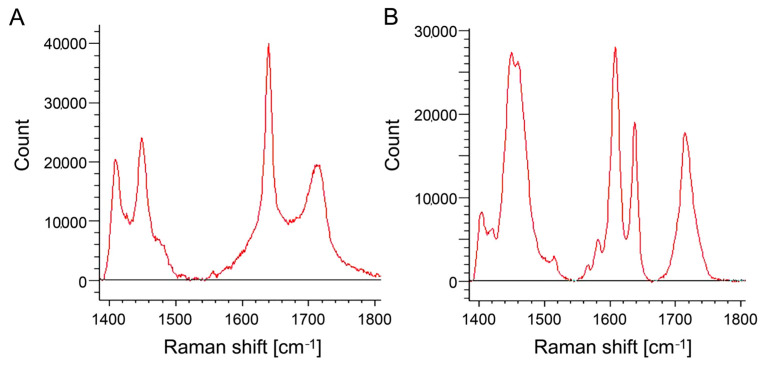
Raman spectra of (**A**) METAC aqueous solution and (**B**) poly(METAC) in EX.

**Figure 6:**
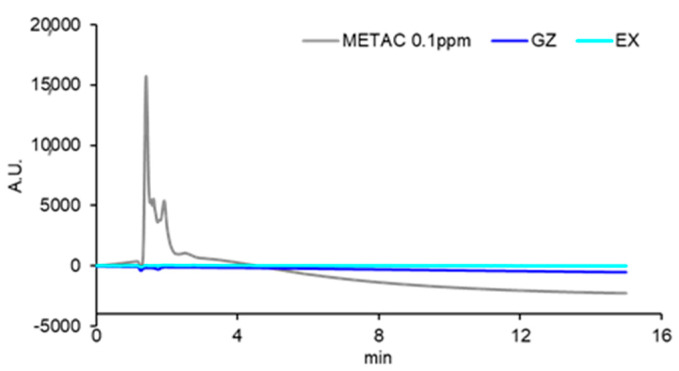
HPLC spectra of GZ and EX eluates. METAC aqueous solution (0.1 ppm) is shown for reference.

Figure 3 shows the SEM image of the surface of the cured EX. On the EX surface, low-intensity (dark) regions with a size of 4.6 ± 1.0 µm were observed. Around the low-intensity regions observed in the FE-SEM image, chlorine, as a counter ion of METAC, was densely distributed in EX in the elemental mapping of the EDS analysis (Figure 4). Nitrogen derived from METAC was not detected in the EX. Figure 5 shows the Raman spectra of the METAC aqueous solution and poly(METAC) in the experimental resin composites. Raman spectroscopy analysis indicated that the polymerization rate of METAC was 46.9 ± 4.0%. No unpolymerized METAC monomers were detected in the cured experimental resin via HPLC analysis (Figure 6).

### 3.2. Antibacterial Activity of Resin Composites Incorporating Poly(METAC)

**Figure 7:**
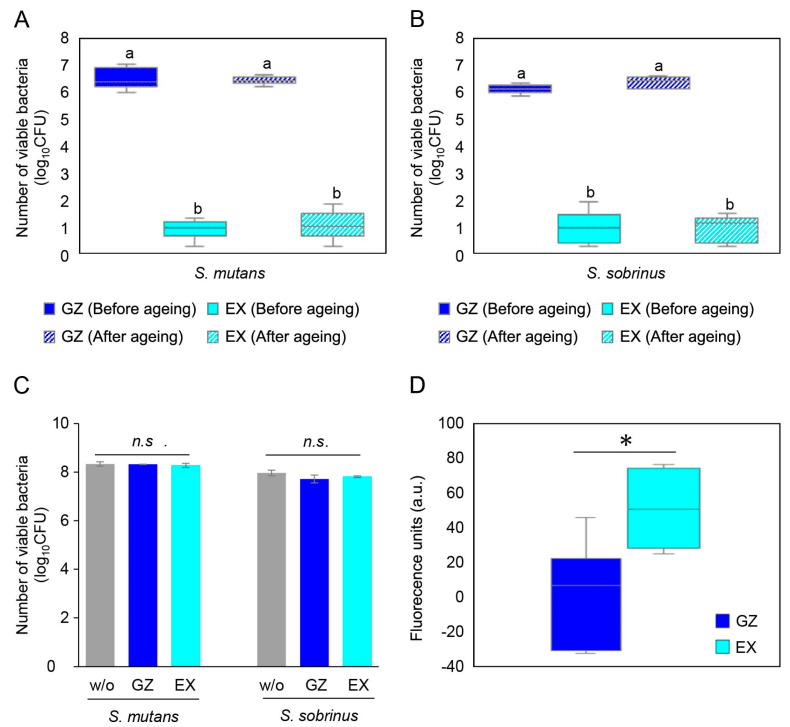
Number of (**A**) *S. mutans* cells or (**B**) *S. sobrinus* cells on the GZ or EX discs after 24 h of incubation. The hatched bar represents the number of bacterial cells on each disc after 28 days of immersion. Error bars represent the standard deviation. a, b: Identical letters indicate no significant difference between the bars (ANOVA, Tukey’s HSD test, *p* > 0.05, n = 6). (**C**) The number of viable *S. mutans* and *S. sobrinus* cells after 24 h incubation in the presence of the eluate from EX. *n.s.*: No significant difference (ANOVA, Tukey’s HSD test, *p* > 0.05, n = 3). (**D**) The fluorescence intensity of DiSC3(5) after incubation in the presence of each specimen. *: *p* < 0.05, Student’s *t*-test, n = 6.

The numbers of viable *S. mutans* and *S. sobrinus* cells after 24 h incubation on the specimens before and after ageing are shown in Figure 7A and 7B, respectively. The number of *S. mutans* and *S. sobrinus* cells on GZ increased from the initial bacterial number (3.70 log_10_CFU) to 6.55 log_10_CFU and 6.16 log_10_CFU, respectively. The numbers of *S. mutans* and *S. sobrinus* cells on EX were reduced to 0.87 log_10_CFU and 0.96 log_10_CFU, respectively. For both *S. mutans* and *S. sobrinus*, the number of surviving cells in EX was significantly lower than those on GZ (*p* < 0.05, ANOVA, Tukey’s HSD test, n = 6). After 28 days of immersion in distilled water, the antibacterial effect of EX was not attenuated, as shown by the lack of a significant difference in the number of *S. mutans* and *S. sobrinus* cells before and after ageing (*p* < 0.05, ANOVA, Tukey’s HSD test, n = 6).

The number of viable *S. mutans* and *S. sobrinus* cells on EX after 24 h of incubation in the presence of the eluate is shown in Figure 7C. No significant difference was observed between the bacterial numbers in the presence and absence of eluates from EX (*p* > 0.05, ANOVA, Tukey’s HSD test, n = 3).

The fluorescence intensity of DiSC_3_(5) after incubation is shown in Figure 7D. The fluorescence intensity increased significantly in the presence of EX compared to GZ (*p* < 0.05, Student’s *t*-test, n = 6).

### 3.3. Physical Properties of Resin Composites Incorporating Poly(METAC)

**Figure 8:**
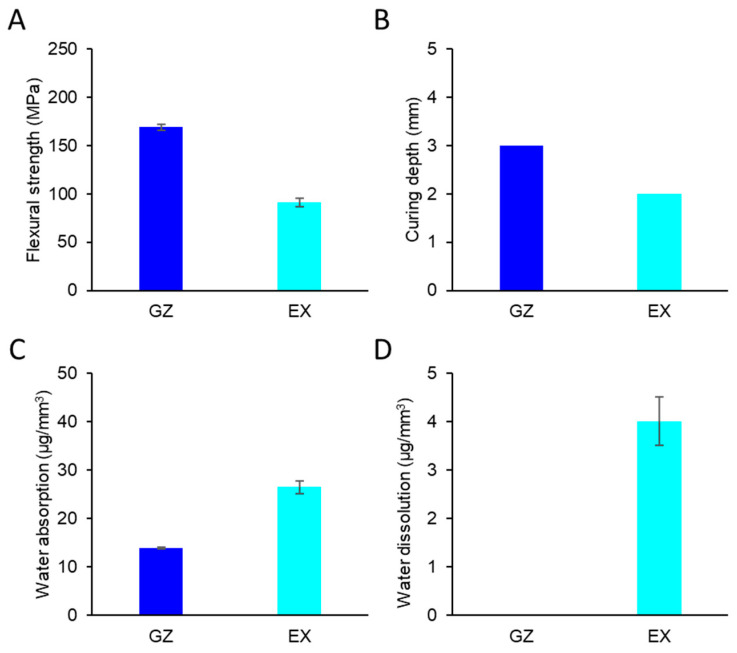
The physical properties of EX and GZ (n = 3). Error bars represent the standard deviation. (**A**) Flexural strength. (**B**) Curing depths. (**C**) Water absorption. (**D**) Water dissolution.

Figure 8 and Table 2 summarize the physical properties of EX and GZ. The flexural strength of EX was lower than that of GZ but was within the ISO-stipulated range. The curing depth, water absorption, and dissolution of EX fulfilled the requirements described in ISO 4049:2019.

## 4. Discussion

One approach to confer antibacterial effects to resin-based dental materials is the immobilization of antibacterial components in/on the materials. This technology utilizes QAC-based resin monomers that can copolymerize with methacrylate resins and achieve antibacterial effects through their quaternary ammonium moieties [1,2,3,12]. Many types of QAC-based monomers, such as MDPB [3,6], methacryloxylethyl cetyl dimethyl ammonium chloride (DMAE-CB) [13], 2-methacryloxylethyl dodecyl methyl ammonium bromide (MAE-DB) [14], bis(2-methacryloyloxyethyl) dimethylammonium bromide (IDMA-1) [15], dimethylaminohexadecyl methacrylate (DMAHDM) [16], urethane dimethacrylates quaternary ammonium methacrylate (UDMQA) [17], and quaternary ammonium bis-phenol A glycerolate dimethacrylate (QABGMA) [18], have been developed. Moreover, advances in nanotechnology have enabled the development of antibacterial dental resins using QAC-functionalized nanofillers. QPEI nanoparticles exhibit strong antibacterial effects against Gram-positive and Gram-negative bacteria, and their antimicrobial potency is attributed to the abundance of quaternary ammonium groups along the polymer backbone [5,19]. Because of their small size and extensive surface area, the addition of a small amount of QAC nanoparticles was enough to provide antibacterial effects, and incorporating 1–2 wt.% ensured contact-killing effects on various materials [20,21,22]. Beyth et al. reported the effect of experimental resin composites containing QPEI nanoparticles in inhibiting biofilm formation based on an in situ study using a custom-made removable acrylic appliance [5]. Recent studies have focused on the surface modification of quaternary ammonium-silica-based nanoparticles [23,24].

Poly(METAC) is a methacrylate-type cationic polymer composed of QAC. METAC monomers can be polymerized alone or copolymerized with other types of methacrylate monomers [7,25,26]. In this study, experimental resin composites were prepared by adding an aqueous METAC solution to a commercial resin-composite paste before curing. FE-SEM/EDS analysis showed that the counter ion (Cl) of METAC was densely distributed in some regions, indicating that METAC was assembled in the resin composites. N is a specific atom derived from METAC in the experimental resin composites. However, distinguishing N with EDS analysis was difficult because the characteristic X-ray wavelength of N is close to that of C. Raman spectroscopy and HPLC analyses confirmed that METAC was sufficiently polymerized in the resin composites. 

MDPB is an antibacterial QAC-based resin monomer that was first reported as a polymerizable bactericide for dental resins [1,3]. The antibacterial effects of various resin-based restorative materials incorporating the QAC-based resin monomer MDPB have been the subject of intensive research over the past two decades. Previous studies reported that resin composites containing MDPB in a matrix at 34.4 wt.% and those containing MDPB-immobilized filler (final concentration at 2.83 wt.%) exhibited a bacteriostatic effect; however, they could not exhibit a bactericidal effect [4,27]. The same method (an on-disc culture assay) was used to evaluate the antibacterial effects of resin composites incorporating poly(METAC) and compare them with those of MDPB-containing resin composites. This study used a higher concentration of bacteria (2 × 10^5^ CFU/mL) to imitate severe conditions in the oral environment. For the resin composites incorporating poly(METAC), the log reductions in *S. mutans* and *S. sobrinus* were 2.83 and 2.74 from their initial numbers, indicating a bactericidal effect rather than a bacteriostatic effect owing to the resin composites incorporating poly(METAC). The antibacterial activities of the resin composites incorporating poly(METAC) after ageing in distilled water for 28 days were at the same level as those before ageing. 

The methacryloyl group in poly(METAC) enabled copolymerization with other monomers. The antibacterial component was immobilized in the resin matrix after polymerization, and poly(METAC) was responsible for the antibacterial activity. The eluate obtained via the immersion of the resin composites incorporating poly(METAC) in distilled water for 28 days did not demonstrate an antibacterial effect against *S. mutans* o *S. sobrinus*. This result indicated that the immobilized antimicrobial component did not leach out in a wet environment. Therefore, cured resins incorporating poly(METAC) could demonstrate contact inhibition, that is, the inhibition of bacteria that come into contact with their surfaces, without releasing any active components. The previous literature on poly(METAC) has demonstrated its non-cytotoxic nature [28,29]. Furthermore, in this study, poly(METAC) was incorporated into commercially available resin composites known for their non-cytotoxicity. Nevertheless, the impact of incorporating poly(METAC) on the biocompatibility of the resin composites needs to be verified.

DiSC_3_(5) is a fluorescent cyanine dye that penetrates cell membranes [30]. Outside the cell, its fluorescence intensity is low. However, when it enters a cell and binds to the cytoplasmic membrane, its fluorescence intensity increases [31]. In the polarized membrane, the dye was in an aggregated state, resulting in low fluorescence. However, when the membrane became depolarized, the dye dispersed within it, leading to an increase in the fluorescence intensity. The fluorescence intensity of DiSC_3_(5) indicated that the immobilized component of EX depolarized the cell membrane. When depolarization occurred at the initial stage of contact, cell viability was reduced via bactericidal–membrane interactions [32]. 

To investigate the influence of METAC on the physical properties of the resin composites, the flexural strength, curing depth, water absorption, and water dissolution of the experimental resin were compared with those of commercially available resin composites without METAC polymer. The curing depth of the experimental resin composites was similar to those of the commercially available resin composites; therefore, the incorporation of poly(METAC) did not affect the absorption spectrum or optical transmittance of the resin composites [33]. The polymerization of METAC was capable of gelling [7], which led to an increase in the water absorption/dissolution of the experimental resin composites. However, the water absorption and dissolution values were within the standards defined by ISO 4049: 2019 [11]. The flexural strength of the experimental resin composites containing poly(METAC) decreased; however, the flexural strength of the resin composites incorporating poly(METAC) after ageing for 7 days was within the ISO-stipulated range. Therefore, the experimental resin composites can potentially be used in clinical applications. We conducted preliminary assessments of METAC at concentrations other than 6% and determined that a 6% concentration is optimal for achieving effective antibacterial effects while maintaining clinically available physical properties. However, further investigations are required to evaluate their physical properties after long-term ageing.

In conclusion, the newly fabricated resin composites containing the QAC-based antibacterial monomer METAC demonstrated acceptable physical properties for clinical use and exhibited long-term bactericidal effects against oral bacteria on its surface. 

## Figures and Tables

**Table 1:** Composition of the materials used in this study.

Material	Code	Composition
GRACEFIL ZeroFlo (GC corporation)	GZ	Dimethacrylate, Silicon dioxide, Barium glass, Pigment, Photo initiator
Experimental resin composites	EX	GRACEFIL ZeloFlo + 6 wt.% METAC

**Table 2:** Physical properties of GZ and EX.

**Property**	**GZ**	**EX**	ISO * range
Flexural strength (MPa)	169 ± 3	91 ± 4	≥80
Curing depth (mm)	3.0	2.0	≥1.5
Water absorption (μg/mm^3^)	13.8 ± 0.2	26.4 ± 1.3	≤40
Water dissolution (μg/mm^3^)	0.0 ± 0.0	4.0 ± 0.5	≤7.5

* ISO: International Organization for Standardization 4049: 2019. Dentistry-polymer-based restorative materials.

## Data Availability

The datasets used and analyzed in the current study are available from the corresponding author upon reasonable request.
